# Structural basis for impairment of DNA methylation by the DNMT3A R882H mutation

**DOI:** 10.1038/s41467-020-16213-9

**Published:** 2020-05-08

**Authors:** Hiwot Anteneh, Jian Fang, Jikui Song

**Affiliations:** Department of Biochemistry, University of California, Riverside, CA 92521 USA

**Keywords:** Transferases, DNA methylation, X-ray crystallography

## Abstract

DNA methyltransferase DNMT3A is essential for establishment of mammalian DNA methylation during development. The R882H DNMT3A is a hotspot mutation in acute myeloid leukemia (AML) causing aberrant DNA methylation. However, how this mutation affects the structure and function of DNMT3A remains unclear. Here we report structural characterization of wild-type and R882H-mutated DNMT3A in complex with DNA substrates with different sequence contexts. A loop from the target recognition domain (TRD loop) recognizes the CpG dinucleotides in a +1 flanking site-dependent manner. The R882H mutation reduces the DNA binding at the homodimeric interface, as well as the molecular link between the homodimeric interface and TRD loop, leading to enhanced dynamics of TRD loop. Consistently, in vitro methylation analyses indicate that the R882H mutation compromises the enzymatic activity, CpG specificity and flanking sequence preference of DNMT3A. Together, this study uncovers multiple defects of DNMT3A caused by the R882H mutation in AML.

## Abstract (web summary)

The DNA methyltransferase DNMT3A plays an important role in establishing the DNA methylation patterns during development and deregulation of DNMT3A is associated with hematological cancers, with the R882H mutation the most frequently occurring DNMT3A missense mutation in acute myeloid leukemia. Here, the authors present the crystal structures of wild-type and R882H DNMT3A in complex with different DNA substrates and explain why the R882H mutation compromises the enzymatic activity of DNMT3A.

## Introduction

DNA methylation is an important epigenetic mechanism that critically impacts cell proliferation and lineage commitment during development^1,2^. In mammals, DNA methylation mainly occurs at the C-5 position of cytosines within CpG dinucleotides, accounting for 70–80% of all CpG sites in the genome^3^. Mammalian DNA methylation is established by de novo DNA methyltransferases DNMT3A and DNMT3B^4^, and subsequently subjected to maintenance by DNA methyltransferase DNMT1 in a replication-dependent manner^5^. DNMT3A-mediated DNA methylation is further regulated by DNMT3-like protein (DNMT3L) during gametogenesis and embryogenesis^6–8^. Dysregulation of DNA methylation is associated with various human diseases^9^, such as cancers^10,11^. Among these, mutation of DNMT3A has been identified in ~25% acute myeloid leukemia (AML) patients^12–15^, which gives rise to aberrant DNA methylation patterns and increased cell proliferation^16–18^, and correlates with poor clinical outcome^13^.

DNMT3A R882H mutation represents the most frequent DNMT3A missense mutation in AML^13^. Previous studies have demonstrated that this mutation leads to CpG DNA hypomethylation and altered gene regulation in hematopoietic or embryonic stem (ES) cells^16,17,19^. Consistently, it has been reported that the R882H mutation impairs the enzymatic activity of DNMT3A^16,17,20–24^, and destabilizes its tetrameric form in vitro and in cells^17,22,23^. Furthermore, recent studies have suggested that the R882H mutation may affect the relative enzymatic preference of DNMT3A toward different CpG-flanking sequences: in comparison with wild-type DNMT3A, the R882H mutant shows stronger preference for the CG(G/A) motif over the CG(T/C) motif as substrate^25^, which correlates with the aberrant DNA methylation and gene expression in AML, suggesting an off-targeting effect of the DNMT3A R882H mutation that may contribute to AML pathogenesis^21^.

The structures of DNMT3A in complex with DNMT3L have been reported, revealing that DNMT3A dimerizes through two alternative interfaces: a DNMT3A–DNMT3A homodimeric interface (a.k.a. RD interface) mediated by polar interactions and a DNMT3A–DNMT3L interface (a.k.a. FF interface) mediated by hydrophobic contacts^24,26,27^. Our recent study on the complex between DNMT3A–DNMT3L and CpG DNA further demonstrated that the DNMT3A–DNA interaction is mediated by three distinct regions: a loop from the target recognition domain (TRD loop), a loop from the catalytic core (catalytic loop) and the RD interface of DNMT3A^24^. Notably, residue R836 from the TRD loop forms a hydrogen bond with the CpG site, which contributes to the CpG specificity of DNMT3A^24^. Structural analysis of the DNMT3A–DNMT3L–DNA complex also confirmed a role of R882, located on the RD interface, in DNA binding^24^. In addition, an intramolecular hydrogen bond is formed between residues R882 and S837, establishing a link between the TRD loop and RD interface.

To gain further insights into the context-dependent DNA methylation by DNMT3A and the mutational effect of R882H, we determined the crystal structure of wild-type DNMT3A (DNMT3A^WT^)–DNMT3L tetramer in complex with a CGA motif-containing DNA, as well as R882H-mutated DNMT3A (DNMT3A^R882H^)–DNMT3L tetramer in complex with CGA- and CGT-containing DNAs. Our study reveals that the TRD loop in the DNMT3A^WT^–DNMT3L–CGA DNA complex recognizes the CpG site in a different mechanism than that in the previously reported DNMT3A^WT^–DNMT3L–CGT DNA complex^24^, therefore uncovering the inherent conformational dynamics of the TRD loop. The R882H mutation not only impairs the protein–DNA contact at the RD interface, but also perturbs the molecular interaction between the RD interface and the TRD loop, leading to altered conformational flexibility of the TRD loop and its context-dependent DNA contact. Consistently, in vitro methylation analyses indicate that the R882H mutation impairs both the CpG methylation efficiency and specificity of DNMT3A. Together, these studies uncover a multifaceted defect of DNMT3A caused by the R882H mutation.

## Results

### Structure of the DNMT3A^WT^–DNMT3L–CGA DNA complex

**Fig. 1:**
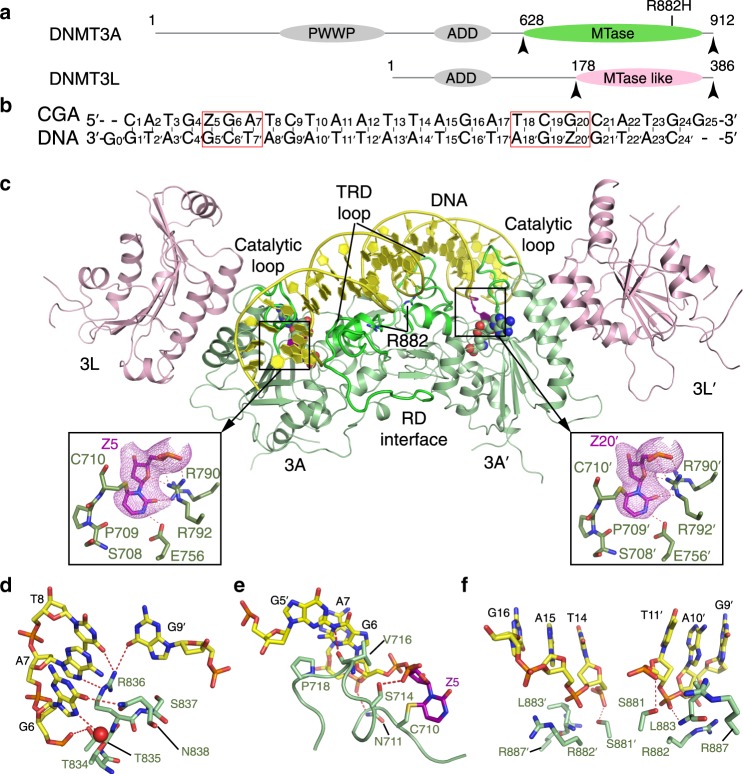
Structure of the DNMT3A^WT^–DNMT3L tetramer in complex with CGA DNA. **a** Domain architecture of DNMT3A and DNMT3L with the C-terminal domains marked with arrowheads. The site for R882H mutation is marked. **b** DNA sequence (CGA) used for the structural study. Z, zebularine. **c** Ribbon representations of DNMT3A^WT^–DNMT3L bound to CGA DNA and SAH, with residue R882 marked. The zebularines anchored at the two active sites are shown in expanded views, with hydrogen-bonding interactions depicted as dashed lines and Fo–Fc omit map (violet) contoured at 2.0 sigma level. The SAH molecules are shown in sphere representation. **d**–**f** Close-up views of the DNA interactions of the TRD loop (**d**), catalytic loop (**e**) and RD interface (**f**) of DNMT3A^WT^.

Our previous study delineated the interaction between DNMT3A^WT^–DNMT3L and DNA containing two CGT motifs^24^. To further investigate how the sequence context of DNA substrates affects DNMT3A-mediated DNA methylation, we set out to determine the crystal structure of DNMT3A^WT^–DNMT3L tetramer in complex with a CGA-containing DNA duplex. In essence, we generated the DNMT3A^WT^–DNMT3L tetramer using the MTase domain of DNMT3A^WT^ and the C-terminal domain of DNMT3L (Fig. 1a). The DNA substrate was prepared by annealing of a self-complimentary 25-mer DNA in which the cytosine within the CGA motif is replaced by a zebularine (Z), a cytosine analogue^28^. The resulting DNA duplex harbors two separate (ZpGpA) • (TpCpG) sites, mimic of two (CpGpA) • (TpCpG) sites (denoted herein as CGA DNA) (Fig. 1b). The crystal structure of the DNMT3A^WT^–DNMT3L–CGA DNA (DNMT3A^WT^–CGA) complex, bound to cofactor byproduct *S*-Adenosyl-L-homocysteine (SAH), was solved by Molecular Replacement using the reported crystal structure of DNMT3A^WT^–DNMT3L–CGT DNA (DNMT3A^WT^–CGT) complex (PDB 5YX2)^24^ as template. The resulting electron density map permitted us to model the entire DNMT3A and DNA molecules. The majority of DNMT3L was also modeled, except for a few loop segments (residues 209–217, 314–317, 354–357 and 380–386). The structure of the DNMT3A^WT^–CGA complex was refined to 2.40 Å resolution (Fig. 1c and Supplementary Table 1).

The structure of the DNMT3A^WT^–CGA complex resembles that of the DNMT3A^WT^–CGT complex (Fig. 1c)^24^, with the zebularines flipped into the active sites of the DNMT3A^WT^ molecules, stabilized via covalent linkage with catalytic cysteine C710 of DNMT3A^WT^ and hydrogen-bonding interactions with other catalytic residues (Fig. 1c). The protein–DNA interaction is mainly mediated by the TRD loop (residues 831–848), catalytic loop (residues 707–721) and a segment at the RD interface (residues 881–887) of DNMT3A^WT^ (Fig. 1c and Supplementary Fig. 1a, b). The TRD loop extends into the DNA major groove, with residues T834 and N838 engaging water-mediated and direct hydrogen-bonding interactions with the N7 and O6 atoms of G6, respectively (Fig. 1d). Meanwhile, residue T835 forms a hydrogen bond with the backbone phosphate of G6, and the side chain of residue R836 approaches the +1 – +3 flanking nucleotides (A7-T8 and G9′) of the CpG site, donating the guanidinium group for hydrogen-bonding interactions with A7 and T8 of the target strand and G9′ of the non-target strand (Fig. 1d). Toward the minor groove, the catalytic loop interacts with the unpaired CpG guanine (G5′/G20) and surrounding DNA backbone through hydrogen-bonding interactions, as well as stacking contacts between residues V716 and P718 and ZpG guanine (G6/G19′) (Fig. 1e). In addition, residues S881, R882, L883, and R887 at the RD interface interact with the DNA backbone (T14-G16 and G9′-T11′) through hydrogen bonding, electrostatic or van der Waals contacts (Fig. 1f). Together, these interactions form three-pronged contacts between DNMT3A dimers and DNA, resulting in a buried surface area of ~2800 Å^2^. On the other hand, DNMT3L is not involved in any contact with DNA, as observed previously^24^.

### Structural comparison of DNMT3A^WT^–CGA and DNMT3A^WT^–CGT

**Fig. 2:**
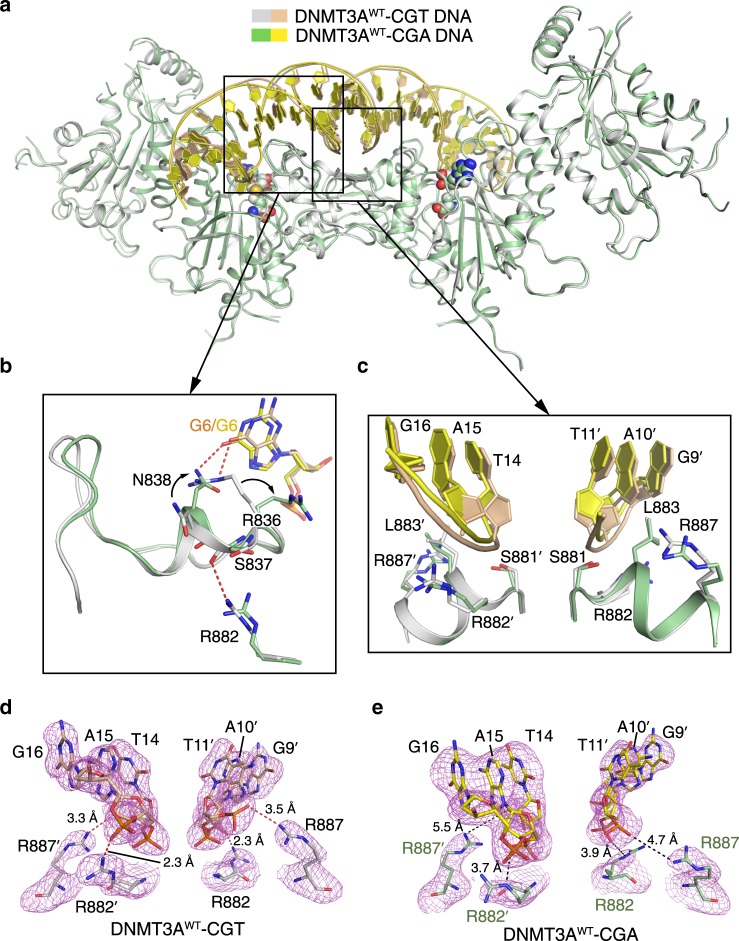
Structural comparison of the DNMT3A^WT^–CGA and DNMT3A^WT^–CGT complexes. **a** Structural superposition of the DNMT3A^WT^–CGA and DNMT3A^WT^–CGT (PDB 5YX2) complexes. **b** Structural comparison of the TRD loop and its interactions with CpG guanine (G6) and R882 in the DNMT3A^WT^–CGA and DNMT3A^WT^–CGT complexes. The conformational rearrangements of the TRD residues between the two complexes are indicated by arrows. **c** Structural comparison of the RD interface between the DNMT3A^WT^–CGA and DNMT3A^WT^–CGT complexes. **d,e** Close-up views of the R882- and R887-engaged DNA interactions in the DNMT3A^WT^–CGT (PDB 5YX2) (**d**) and DNMT3A^WT^–CGA (**e**) complexes, with the Fo–Fc omit map (violet) contoured at 2.0 sigma level. The distances for hydrogen-bonding and electrostatic interactions are measured and indicated by dashed lines, colored in red and black, respectively.

Structural comparison of DNMT3A^WT^–CGA with DNMT3A^WT^–CGT reveals high structural similarity over the entire complex, with root-mean-square deviation (RMSD) of 0.42 Å over 821 aligned Cα atoms (Fig. 2a). Nevertheless, the DNMT3A^WT^–CGA complex differs considerably from the DNMT3A^WT^–CGT complex in two regions: the TRD loop and the RD interface (Fig. 2b,c). In the DNMT3A^WT^–CGT complex, the side chain of residue R836 on the TRD loop forms a base-specific hydrogen bond with G6, while the neighboring N838 forms a hydrogen bond with the DNA backbone (Supplementary Fig. 1c); in contrast, in the DNMT3A^WT^–CGA complex, the side chain of R836 flips away from G6 to interact with the +1 – +3 flanking nucleotides through hydrogen-bonding interactions; meanwhile, the side chain of N838 turns to occupy the space vacated by the flipped R836, thereby engaging a base-specific hydrogen-bonding interaction with G6 (Fig. 2b and Supplementary Fig. 1d). Such a conformational rearrangement in the TRD loop also perturbs the interaction between the RD interface and the TRD loop (Fig. 2b): the hydrogen bond formed between the side chain of R882 and the backbone carbonyl of S837 in the DNMT3A^WT^-CGT complex becomes disrupted in the DNMT3A^WT^–CGA complex (Fig. 2b). Given that our previous study indicated that the TRD loop undergoes a disorder-to-order transition upon substrate binding, with the substrate-bound form partially stabilized by the interaction between the TRD loop and RD interface^24^, we surmised that loss of the hydrogen bond between S837 and R882 in the DNMT3A^WT^–CGA complex would presumably lead to enhanced flexibility of the TRD loop. Indeed, B-factor analysis of the two DNMT3A^WT^-DNA complexes indicates that whereas residues R836-N838 in the DNMT3A^WT^–CGT complex are associated with an averaged B-factor (66.5 Å^2^), which is comparable with the overall averaged B-factor of DNMT3A (62.5 Å^2^), the same region in the DNMT3A^WT^–CGA complex is associated with an averaged B-factor (87.7 Å^2^) significantly higher than the overall averaged B-factor (70.9 Å^2^), supporting the enhanced flexibility of the TRD loop in the DNMT3A^WT^–CGA complex.

In line with the perturbation of the interaction between the TRD loop and RD interface, structural comparison of the DNMT3A^WT^–CGA and DNMT3A^WT^–CGT complexes also reveals a subtle conformational change in the RD interface (Fig. 2c). In the DNMT3A^WT^–CGT complex, residues S881, R882, L883, and R887 on the RD interface all engage side-chain hydrogen-bonding or van der Waals interactions with DNA backbone (Fig. 2c, d), resulting in a buried surface area of 423 Å^2^. By contrast, in the DNMT3A^WT^–CGA complex, although residues S881 and L883 retain the same fashion of DNA interactions, the other two residues, R882 and R887, are no longer within a distance for hydrogen bond formation with the DNA backbone (Fig. 2c, e), leading to a reduced buried surface area (289 Å^2^) in this region.

Together, these observations suggest that the CpG sites with different flanking sequences may result in different CpG-recognition modes of the TRD loop, as well as perturbation of the interaction between the TRD loop and the RD interface.

### Structure of the DNMT3A^R882H^–CGA DNA complex

**Fig. 3:**
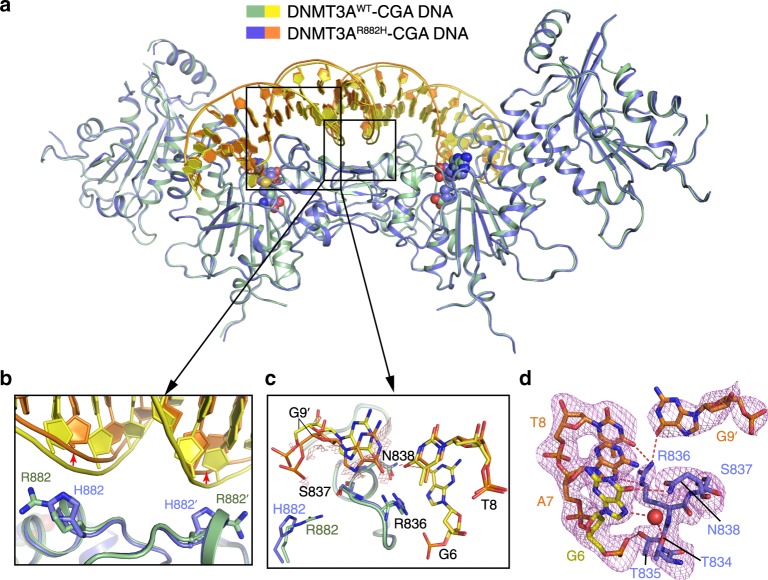
Structural comparison of the DNMT3A^WT^–CGA and DNMT3A^R882H^–CGA complexes. **a** Structural superposition of the DNMT3A^WT^–CGA and DNMT3A^R882H^–CGA complexes. **b** Structural comparison of the RD interface between the DNMT3A^WT^–CGA and DNMT3A^R882H^–CGA complexes. **c** Structural comparison of the TRD loop and its interactions with CpG guanine (G6) and R882 in the DNMT3A^WT^–CGA and DNMT3A^R882H^–CGA complexes. The hydrogen-bonding interactions are indicated by dashed lines. The conformational difference of G9′ is validated by the Fo–Fo difference map (salmon) between the two complexes, contoured at 1.0 sigma level. **d** Close-up views of the DNA interactions of the TRD loop in the DNMT3A^R882H^–CGA complex, with the Fo–Fc omit map (violet) contoured at 2.0 sigma level. The water molecule is shown as a red sphere.

To understand how the R882H mutation of DNMT3A affects its structure and function, we determined the crystal structure of the complex between the DNMT3A^R882H^–DNMT3L tetramer and CGA DNA (DNMT3A^R882H^–CGA), containing the same sequence as that for the DNMT3A^WT^–CGA complex, and SAH at 2.5 Å resolution (Fig. 3a and Supplementary Table 1). Structural alignment of the entire DNMT3A^R882H^-CGA complex with the DNMT3A^WT^–CGA complex gives an RMSD of 0.29 Å over 875 aligned Cα atoms, suggesting that the R882H mutation does not alter the structural integrity of the DNMT3A–DNMT3L tetramer (Fig. 3a). The most notable structural difference between the DNMT3A^WT^–CGA and DNMT3A^R882H^–CGA complexes lies in the DNA conformation: In comparison with the DNMT3A^WT^–CGA complex, the central segment of the DNMT3A^R882H^-bound DNA molecule bends away by ~2 Å from the RD interface in the DNMT3A^R882H^–CGA complex (Fig. 3b and Supplementary Fig. 2a). The protein–DNA contact at the RD interface of DNMT3A^R882H^ in the CGA DNA complex is mainly mediated by residues S881, H882, and L883, which form hydrogen bonds and/or van der Waals contacts with the DNA backbone (Supplementary Fig. 2b,c). The fact that the DNMT3A^R882H^–CGA complex was crystalized under acidic condition (pH 4.2; see Methods) likely contributed to the H882-mediated hydrogen-bonding interaction. In addition, the side chain of R887 is poised for electrostatic interaction with the backbone phosphate of A15/A10′ (Supplementary Fig. 2c). Together, these interactions lead to a buried surface area of 238 Å^2^ in this region, lower than that for the DNMT3A^WT^–CGA complex, suggesting that the R882H mutation leads to reduced protein–DNA contact at the RD interface.

The TRD loop in the DNMT3A^R882H^–CGA complex adopts a similar conformation to that in the DNMT3A^WT^–CGA complex, with residue N838 engaging a base-specific hydrogen-bonding interaction with G6, and R836 forming hydrogen bonds with the +2 (T8) and +3 (G9′) flanking bases (Fig. 3c, d). Nevertheless, subtle conformational shift was observed for G9′, likely resulting from the altered DNA curvature (Fig. 3c). In addition, residues T834 and T835 both engage DNA contacts in the same fashion as their counterparts in the DNMT3A^WT^–CGA complex (Fig. 3c). Furthermore, as with the DNMT3A^WT^–CGA complex, the hydrogen bond between the side chain of R882 and the backbone carbonyl of S837 is also abrogated in the DNMT3A^R882H^–CGA complex (Fig. 3c). Accordingly, residues R836-N838 are associated with a significantly higher averaged B-factor (80.7 Å^2^) than that averaged for the entire DNMT3A^R882H^ molecule (65.2 Å^2^), indicating the significant conformational dynamics of the TRD loop in the DNMT3A^R882H^–CGA complex.

### Crystal structure of the DNMT3A^R882H^–CGT DNA complex

**Fig. 4:**
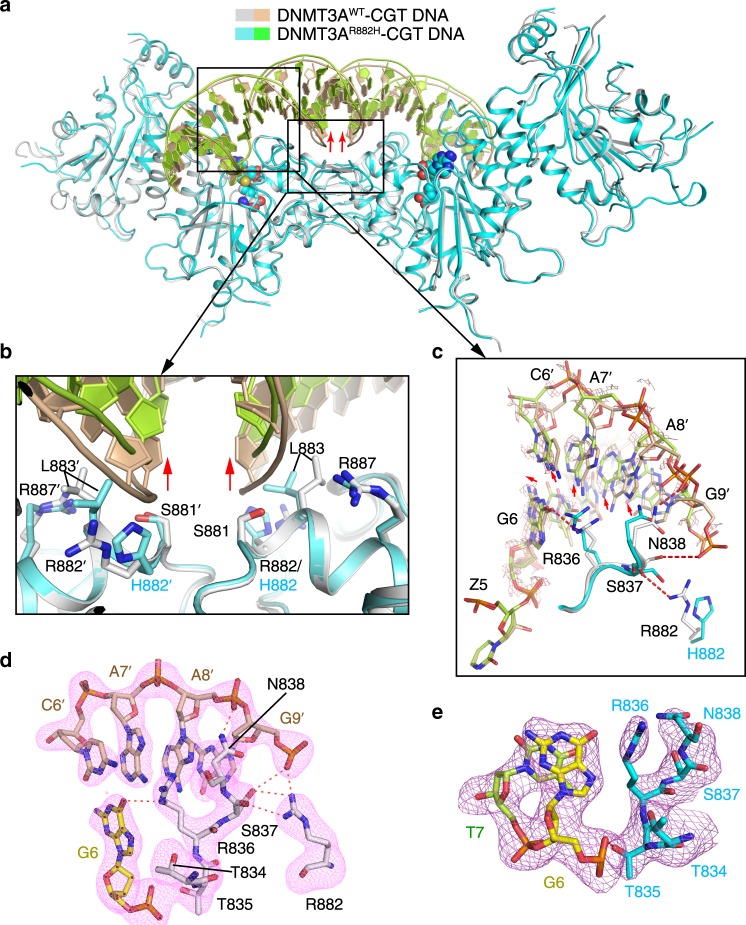
Structural comparison of the DNMT3A^WT^–CGT and DNMT3A^R882H^–CGT complexes. **a** Structural superposition of the DNMT3A^WT^–CGT (PDB 5YX2) and DNMT3A^R882H^–CGT complexes. **b** Close-up view of protein–DNA interactions on the RD interface of the DNMT3A^WT^–CGT and DNMT3A^R882H^–CGT complexes. **c** Close-up view of the TRD loop and its interactions with CpG guanine (G6) and R882 in the DNMT3A^WT^–CGT and DNMT3A^R882H^–CGT complexes in expanded view. The hydrogen-bonding interactions are indicated by dashed lines. The conformational shifts of the DNA between the two complexes are validated by the 1.0 sigma Fo–Fo difference map (salmon) between the two complexes and indicated by arrows. **d, e** Close-up view of the interactions between the TRD loop and DNA in the DNMT3A^WT^–CGT (PDB 5YX2) (**d**) and DNMT3A^R882H^–CGT (**e**) complexes. The Fo–Fc omit map (violet) for the corresponding regions were contoured at 2.0 sigma level.

Next, to elucidate how the R882H mutation influences the context-dependent DNA methylation by DNMT3A, we characterized the structure of the DNMT3A^R882H^–DNMT3L tetramer in complex with SAH and CGT-containing DNA (DNMT3A^R882H^–CGT), with the same sequence as that previously used for crystalizing the DNMT3A^WT^–CGT complex^24^ (Supplementary Fig. 3a). The structure of the DNMT3A^R882H^–CGT complex was solved at 2.6 Å resolution (Fig. 4a and Supplementary Table 1), well aligned with that of the DNMT3A^WT^–CGT complex, with an RMSD of 0.37 Å over 849 aligned Cα atoms (Fig. 4a).

Structural comparison of the DNMT3A^R882H^–CGT and DNMT3A^WT^–CGT complexes again reveals a difference in the DNA conformation: relative to the DNMT3A^WT^-bound DNA, the DNMT3A^R882H^-bound DNA moves away from the RD interface by ~3 Å, reflective of reduced protein–DNA contact (Fig. 4a). Indeed, protein residues on the RD interface engage much reduced protein–DNA contacts in the DNMT3A^R882H^–CGT complex than the corresponding region in the DNMT3A^WT^–CGT complex (Fig. 4b and Supplementary Fig. 3b,c). Of particular note, the side chains of residues H882 and R887 in the DNMT3A^R882H^–CGT complex are positioned further away from the DNA backbone than their counterparts in the DNMT3A^WT^–CGT complex (Fig. 4b and Supplementary Fig. 3c). As a result of these structural changes, protein–DNA contact at the RD interface in the DNMT3A^R882H^–CGT complex accounts for a buried surface area of ~206 Å^2^ only, much less than that for the DNMT3A^WT^–CGT complex.

The molecular environment for the TRD loop between the DNMT3A^R882H^–CGT and DNMT3A^WT^–CGT complexes also appears distinct: First, as in the DNMT3A^R882H^–CGA complex, the intramolecular hydrogen bond formed between residues S837 and R882 in the DNMT3A^WT^–CGT complex becomes abolished in the DNMT3A^R882H^–CGT complex (Fig. 4c). Second, the DNA molecules in the DNMT3A^R882H^–CGT and DNMT3A^WT^–CGT complexes show differential extent of helical twist (Fig. 4c): In comparison with the DNMT3A^WT^–CGT complex, the DNA major groove in the DNMT3A^R882H^–CGT complex is positioned further away from the TRD loop (Fig. 4c). In line with these environmental changes, the DNMT3A^R882H^–CGT and the DNMT3A^WT^–CGT complex manifests distinct dynamics on the TRD loop: the electron density for the side chains of R836 and N838 in the DNMT3A^R882H^–CGT complex becomes either barely traceable or significantly reduced than their counterpart in the DNMT3A^WT^–CGT complex (Fig. 4d,e). Consistently, the averaged B-factor for residues R836-N838 (119.8 Å^2^) is significantly higher than the overall averaged B-factor (89.9 Å^2^) of DNMT3A^R882H^ in the DNMT3A^R882H^–CGT complex. It is worth noting that the overall B-factor of DNMT3A^R882H^ in the DNMT3A^R882H^–CGT complex is also much higher than that of DNMT3A^WT^ in the DNMT3A^WT^–CGT complex (89.9 Å^2^ vs 62.5 Å^2^), despite their similar resolution and crystallization packing. Nevertheless, such structural and dynamic changes between the two complexes reinforce the notion on the interplay between the dynamics of TRD loop and DNA binding^24^, and suggest an impairment of CpG recognition in the DNMT3A^R882H^–CGT complex.

### Structural analysis of the DNMT3A^R882H^–CAG DNA complex

**Fig. 5:**
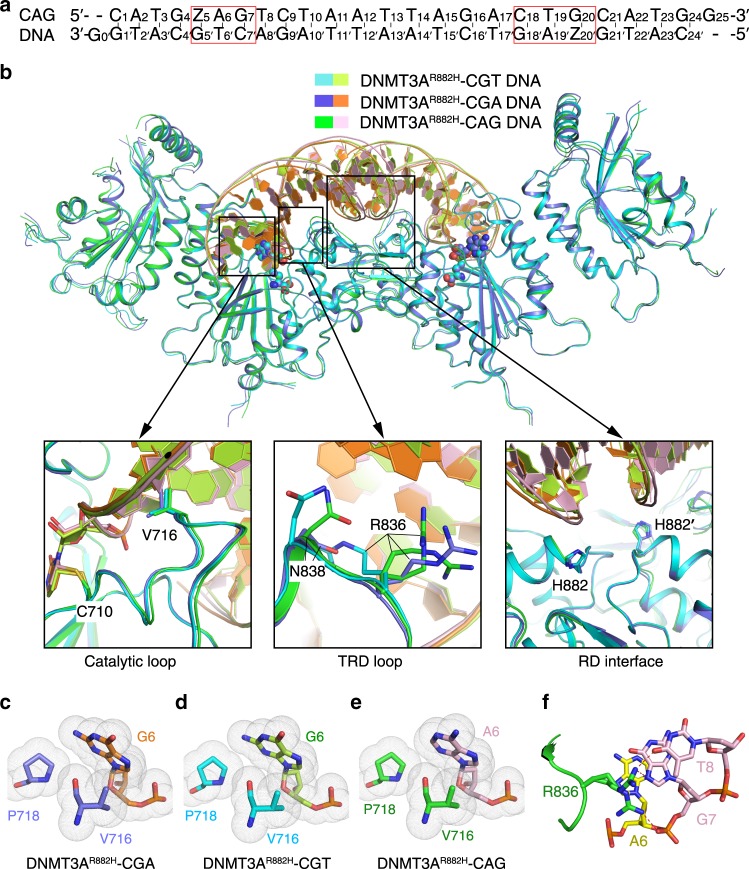
Structural analysis of the DNMT3A^R882H^–CAG complex. **a** DNA sequence (CAG) used for the structural study. **b** Structural superposition of the DNMT3A^R882H^–CGA, DNMT3A^R882H^–CGT and DNMT3A^R882H^–CAG complexes, with the aligned catalytic loops, TRD loops and RD interfaces shown in expanded views. The side chains of the DNA-contacting residues are shown in stick representation. **c**–**e** van der Waals contacts between the catalytic loop (V716 and P718) and the nucleotide downstream of the methylation site (G6 or A6) in the DNMT3A^R882H^–CGA (**c**), DNMT3A^R882H^–CGT (**d**) and DNMT3A^R882H^–CAG (**e**) complexes. **f** Close-up views of the interaction between TRD residue R836, with two alternative conformations, and the CAG DNA. The hydrogen-bonding interactions are shown as dashed lines.

It has been established that DNMT3A not only mediates CpG methylation, but also catalyzes non-CpG methylation, with the enzymatic preference following an order of CpG > CpA > CpT^29–33^. We therefore asked how the conformation of the TRD loop of DNMT3A^R882H^ interplays with non-CpG DNA. To address this question, we determined a 2.45 Å-resolution crystal structure of DNMT3A^R882H^–DNMT3L tetramer in complex with SAH and a 25-mer DNA duplex containing two separate ZpApG sites, mimic of two CpApG sites (Fig. 5a,b and Supplementary Table 1). Structural alignment of the entire DNMT3A^R882H^–DNMT3L–CAG DNA (DNMT3A^R882H^–CAG) complex with the DNMT3A^R882H^–CGT and DNMT3A^R882H^–CGA complexes gives an RMSD of 0.40 Å and 0.41 Å over 845 an 827 aligned Cα atoms, respectively, highlighting their structural similarity (Fig. 5b). Consistently, structural comparison of the three DNMT3A^R882H^–DNA complexes reveals similar DNA binding sites, formed by the catalytic loop, TRD loop and the RD interface (Fig. 5b). In particular, the catalytic loop in the DNMT3A^R882H^–DNMT3L–CAG DNA complex is well aligned with the corresponding region in the CGT and CGA DNA complexes, with residue V716 moving into the DNA cavity vacated by the base flipping of zebularine to stack against the ZpA adenosine (A6) (Fig. 5b and Supplementary Fig 4b,c). Nevertheless, due to the different base ring sizes, A6 in the CAG complex engages less van der Waals contact with the catalytic loop residues (V716 and P718) than the corresponding G6 in the CGA and CGT complexes (Fig. 5c–e), which may contribute to the reduced methylation efficiency of DNMT3A on CpA over CpG. Furthermore, the RD interface is also well aligned among the three complexes, with the side chain of residue H882 poised in a similar conformation (Fig. 5b). Accordingly, the protein–DNA contact at the RD interface of the DNMT3A^R882H^–CAG complex leads to a buried surface area of ~207 Å^2^, comparable with that in the DNMT3A^R882H^–CGT and DNMT3A^R882H^–CGA DNA complexes. The structural similarity between the CpA and CpG DNA complexes of DNMT3A^R882H^ indicate that DNMT3A mediates CpA methylation in a similar fashion as it does for the CpG methylation, highlighting its conserved catalytic mechanism in DNA methylation, as well as the structural rigidity of the DNMT3A^R882H^–DNMT3L tetramer.

Similar to what was observed for the DNMT3A^R882H^–CGA complex, the side chain of R836 points toward the +1 – +2 flanking nucleotides, albeit with two alternative conformations: one R836 conformer approaches the O4 atom of T8 for hydrogen bond formation, while the other points toward the backbone phosphate of G7 to form a salt bridge (Fig. 5b,f). However, unlike the DNMT3A^R882H^–CGA DNA complex in which N838 forms base-specific hydrogen bond with CpG guanine, the equivalent residue in the DNMT3A^R882H^–CAG complex is not involved in any contact with DNA (Fig. 5b and Supplementary Fig. 4d). These observations further suggest the conformational flexibility of the TRD loop of DNMT3A^R882H^ in accommodating the DNA substrates in different sequence contexts, and reinforce the notion that enhanced conformational dynamics of the TRD loop by the R882H mutation impairs CpG-specific DNA methylation. It is worth noting that a water-mediated hydrogen bond is formed between the side chain of T834 and the N7 atom of A6 in the DNMT3A^R882H^–CAG complex (Supplementary Fig. 4d), resembling the interaction between T834 and the CpG guanine in the DNMT3A–CpG complexes (Figs. 1d and 3d)^24^. Given the fact that a thymine base lacks the corresponding N7 group, this interaction may underpin the differential methylation efficiency of DNMT3A on CpA- and CpT-containing DNAs.

### Structural integrity of the RD interface

Structural analysis of the three DNMT3A^R882H^–DNA complexes reveals that the RD interface is maintained in the same fashion as the DNMT3A^WT^–DNA complexes, dominated by the reciprocal salt bridges formed between residue R885 from one DNMT3A monomer and residue D876 from the symmetry-related mate (Supplementary Fig. 5a–e). These observations therefore suggest that the RD interface-mediated homodimerization is preserved in DNMT3A^R882H^, consistent with previous biochemical studies that this residue does not directly contribute to the formation of DNMT3A homodimer in the context of the DNMT3A–DNMT3L tetramer^24,26,27^. The caveat of this observation is that it concerns only the structure of DNTM3A^R882H^ complexed with DNMT3L, a regulatory factor that is under-expressed in somatic tissues^8^. Therefore, the effect of the R882H mutation on the oligomerization of DNMT3A in hematopoietic stem cells awaits further investigation.

### DNA-binding and enzymatic analysis of DNMT3A^R882H^

**Fig. 6:**
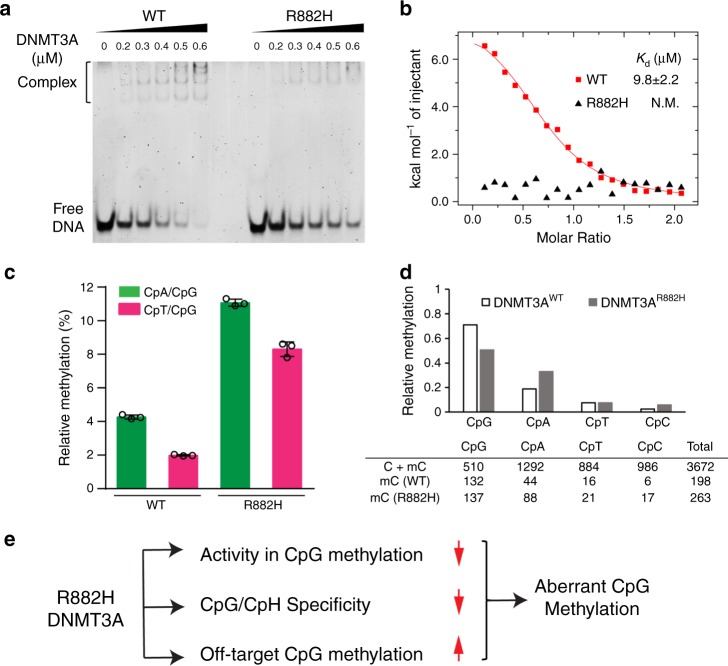
Biochemical and enzymatic analyses of the DNMT3A^R882H^–DNA interaction. **a** EMSA gel image of the DNA binding activities of WT and R882H-mutated DNMT3A–DNMT3L on 36-mer (GAC)_12_ DNA. Source data are provided as a Source Data file. The experiment was repeated once with consistent results. **b** ITC binding analysis of WT and R882H-mutated DNMT3A-DNMT3L on 24-mer (GAC)_8_ DNA. The data and error estimate were derived from two independent experiments. N.M., not measurable. **c** In vitro CpG and CpH methylation of WT and R882H-mutated DNMT3A–DNMT3L, using a 36-mer DNA containing a single CpG, CpA or CpT site (*n* = 3 biological replicates). The data are displayed as ratios of CpA/CpG and CpT/CpG methylation. Data are mean ± s.d. Source data are provided as a Source Data file. **d** (Top) Relative in vitro CpG and CpH methylation of WT and R882H-mutated DNMT3A–DNMT3L on a 626-bp DNA. The data are displayed as ratios of the methylation events of each indicated sequence context over total methylation events. (Bottom) Summary of total cytosine sites and methylation events in each indicated context, derived from bisulfite sequencing analyses of 34 clones of the 626-bp DNA incubated with WT or R882H-mutated DNMT3A–DNMT3L. Source data are provided as a Source Data file. **e** Model for the impairment of CpG methylation by the DNMT3A R882H mutation.

Given that the structures of the DNMT3A^R882H^–DNA complexes suggest that the R882H mutation might lead to impaired DNA contact at both the RD interface and the TRD loop, we next performed biochemical and enzymatic analyses to evaluate the functional consequence of the R882H mutation. First, we compared the DNA binding activities of the DNMT3A^WT^–DNMT3L tetramer and DNMT3A^R882H^–DNMT3L tetramer for a 36-mer CpG DNA using electrophoretic mobility shift assay (EMSA). Consistent with what previously reported^27^, titration of the DNA with the DNMT3A^WT^–DNMT3L tetramer led to the appearance of multiple slow-mobility bands that correspond to the DNMT3A^WT^–DNMT3L–DNA complexes with various stoichiometric ratios (Fig. 6a). By contrast, significantly reduced gel shift was observed for the DNMT3A^R882H^–DNMT3L tetramer (Fig. 6a). Furthermore, we performed isothermal titration calorimetry (ITC) binding assays to compare the binding affinities of DNMT3A^WT^–DNMT3L and DNMT3A^R882H^–DNMT3L for a 24-mer CpG DNA. DNMT3A^WT^–DNMT3L binds to the DNA with dissociation constant (*K*_d_) of 9.8 μM; under the same experimental condition, no appreciable DNA binding was observed for DNMT3A^R882H^–DNMT3L (Fig. 6b). Together, these data confirm that the R882H mutation leads to reduced DNA-binding affinity of DNMT3A.

Next, we performed in vitro enzymatic analysis to evaluate the effect of the R882H mutation on the enzymatic activity and specificity of DNMT3A. Toward this, we measured the enzymatic activities of DNMT3A^WT^–DNMT3L and DNMT3A^R882H^–DNMT3L on CG-, CA- and CT-containing DNAs (Supplementary Fig. 6c–g). Under the experimental condition, the R882H mutation reduced the activity of DNMT3A on CG-containing DNA by 3.3-fold (Supplementary Fig. 6c), consistent with previous observations that this mutation reduces the enzymatic activity of DNMT3A^17,20–22,24^. By contrast, the methylation activity of DNMT3A^R882H^ on CA-containing DNA was reduced by 1.3-fold, and was increased by 1.3-fold on CT-containing DNA. (Supplementary Fig. 6d). As a result, the relative preferences of DNMT3A for CG/CA and CG/CT DNAs were reduced by 2.6- and 4.2-fold, respectively (Fig. 6c). To evaluate the effect of the R882H mutation on the DNMT3A activities toward CpG and non-CpG sites located on the same DNA substrate, we also measured the enzymatic activities of DNMT3A^WT^–DNMT3L and DNMT3A^R882H^–DNMT3L on a 626-bp long DNA substrate, containing multiple CpG, CpA, CpT, and CpC sites. To allow for accurate comparison of CpG specificity, the protein levels and reaction times for DNMT3A^WT^–DNMT3L and DNMT3A^R882H^–DNMT3L were adjusted to achieve a similar level of CpG methylation. After methylation reaction with DNMT3A^WT^–DNMT3L or DNMT3A^R882H^–DNMT3L, the DNA substrates were subjected to bisulfite sequencing analysis. As expected for the high CpG/non-CpG specificity of DNMT3A^24,29^, the DNA substrates methylated by DNMT3A^WT^–DNMT3L show dominant CpG methylation over CpH (H = A, C, or T) methylation (Fig. 6d and Supplementary Fig. 6h). In comparison, the DNA substrates methylated by DNMT3A^R882H^–DNMT3L show more populated methylation events on CpH sites, with significantly increased CpA and CpC methylation (Fig. 6d and Supplementary Fig. 6h). Furthermore, analysis of the relative methylation levels of the CGX (X = G, A, T or C) motif in the DNA substrate also indicates that the R882H mutation shifts the relative methylation efficiencies of DNMT3A^WT^–DNMT3L toward the CG(G/A) sites (Supplementary Fig. 6i), consistent with the observation in a recent report^25^. Together, these data lend a strong support to the structural observation that the R882H mutation results in impaired CpG recognition of DNMT3A.

## Discussion

DNA methylation is an essential epigenetic mechanism that critically regulates gene expression, genomic stability, and cell lineage commitment. Dysregulation of DNA methyltransferases is linked to a variety of human diseases, with DNMT3A mutations particularly implicated in hematological cancers and DNMT3B mutations associated with ICF syndrome^34^. A detailed understanding of the structural and functional consequences of DNMT3A or DNMT3B mutations is vital to deciphering their pathological effect, which will ultimately benefit the development of novel therapeutic strategies against diseases. This study, through structural and biochemical characterizations of DNMT3A^R882H^–DNA interaction, reveals multiple functional impacts of the AML-associated hotspot mutation DNMT3A R882H: The R882H mutation not only reduced the protein–DNA interactions at the RD interface, but also impairs the TRD loop-mediated CpG recognition, which may together contribute to aberrant CpG methylation in AML.

Importantly, this study uncovers the interplay between the intrinsic conformational dynamics of the TRD loop of DNMT3A and its CpG recognition. In particular, the DNMT3A^WT^-CGA complex presented in this study, along with the DNMT3A^WT^–CGT complex we reported previously^24^, reveals that residues R836-N838 of the TRD loop adopt different conformations when binding to DNA substrates with different sequence contexts, highlighting the conformational flexibility of this region. In the DNMT3A^WT^–CGT complex, R836 forms a base-specific hydrogen bond with the O6 atom of CpG guanine, which ensures the CpG specificity, as described previously^24^. However, in the DNMT3A^WT^–CGA complexes, R836 turns away from the CpG site to form a hydrogen bond with the +1A, as well as +2 or +3 flanking nucleotides. Meanwhile, the side chain of N838 turns to the position vacated by the side chain of R836 to form the base-specific hydrogen bond with the O6 atom of CpG guanine, which presumably compensates for the loss of R836-medated CpG recognition. Conceivably, these alternative CpG recognition modes provide a mechanism by which DNMT3A senses the flanking sequence of CpG sites, which may play a role in fine-tuning the DNA methylation patterns across the genome. A full understanding of these alternative CpG recognition modes awaits further investigation.

This study also reveals the crosstalk between the RD interface and the TRD loop of DNMT3A. Introduction of the R882H mutation reduces the interactions between the RD interface and DNA. As a result, the DNA molecule in the DNMT3A^R882H^–DNA complex bends further away from the RD interface, and the hydrogen bond between R882 and S837 becomes disrupted. These structural changes may together contribute to the mobilization of the TRD loop, thereby impairing the context-dependent CpG recognition by DNMT3A. Along the line, the enzymatic preference toward the flanking sequences of CpG target sites has previously been observed for both DNMT3A and DNMT3B^35–38^, yet the mechanism remains unclear. The enzymatic analysis in this study not only supports an earlier notion that the DNMT3A R882H mutation shifts the enzymatic preference of DNMT3A toward a purine (G or A) on the +1 flanking site^25^ (Supplementary Fig. 6d), but also indicates that the R882H mutation reduces the CpG/CpH specificity of DNMT3A, both of which presumably impact the DNA methylation landscape across the genome^25^. Importantly, the crosstalk between the RD interface and the TRD loop provides a mechanism by which the R882H mutation affects the substrate specificity of DNMT3A. It is also conceivable that similar conformational changes in the TRD loop and RD interface can also be induced by the R882C mutation, a second most prominent missense mutation of DNMT3A in AML, and other R882 mutations^13^.

The observation that the DNMT3A R882H mutation impacts the context-dependent DNA methylation suggests this mutation might impair the cellular CpG methylation through multiple pathways (Fig. 6e): (1) the R882H mutation directly reduces the CpG methylation activity of DNMT3A; (2) the R882H mutation may influence the flanking sequence preference of DNMT3A, resulting in aberrant DNA methylation patterns; and (3) the R882H mutation may shift the events of the cytosine methylation toward non-CpG methylation, which would be subjected to immediate elimination during replication-dependent maintenance DNA methylation by DNMT1, an enzyme that shows strict CpG specificity^5,39–42^. Collectively these functional defects of the R882H-mutated DNMT3A might contribute to the aberrant DNA methylation in AML.

## Methods

### Protein expression and purification

DNA fragments encoding the C-terminal domains of WT or R882H-mutated DNMT3A (residues 628-912) and DNMT3L (residues 178-386) were inserted in tandem into pRSFDuet-1 vector (Novagen) containing an N-terminal His_6_-SUMO tag (See Supplementary Table 2 for primer information). The DNMT3A^WT^–DNMT3L and DNMT3A^R882H^–DNMT3L complexes were expressed in *Escherichia coli* BL21 DE3 (RIL) cells, as described previously^24^, followed by purification using a Ni^2+^-NTA affinity column, with buffer A (50 mM Tris-HCl, pH 8.0, 1 M NaCl and 25 mM imidazole) used for sample loading and wash, and buffer B (50 mM Tris-HCl, pH 8.0, 1 M NaCl and 300 mM imidazole) for elution. Upon removing the His_6_-SUMO tag via Ubiquitin-like protease 1 (ULP1)-mediated cleavage the protein complex was further purified via 100–600 mM NaCl gradient on a Heparin column (low salt buffer: 25 mM Tris-HCl, pH 8.0, and 100 mM NaCl; high salt buffer: 25 mM Tris-HCl, pH 8.0, and 1 M NaCl), and size-exclusion chromatography on a 16/600 Superdex 200 pg column (GE Healthcare) in buffer containing 20 mM Tris-HCl (pH 8.0), 100 mM NaCl, 0.1% β-mercaptoethanol and 5% glycerol. The purified DNMT3A^WT^-DNMT3L or DNMT3A^R882H^-DNMT3L tetramer was concentrated to 0.3 mM and stored in −80 °C freezer before use.

### Preparation of enzyme-substrate covalent complexes

25-mer self-complementary DNA sequences were used for generation of the DNMT3A^R882H^–DNMT3L–CGT (CGT DNA: 5′-GCATGZGTTCTAATTAGAACGCATG-3′; Z = zebularine), DNMT3A^WT^–DNMT3L–CGA and DNMT3A^R882H^–DNMT3L–CGA (CGA DNA: 5′-CATGZGATCTAATTAGATCGCATGG-3′; Z = zebularine) and DNMT3A^R882H^–DNMT3L–CAG (CAG DNA: 5′-CATGZAGTCTAATTAGACTGCATGG-3′; Z = zebularine) complexes. The DNA molecules were first subject to a denaturation and annealing process to generate duplexed DNA samples. Subsequently, the DNA was incubated with the DNMT3A^WT^–DNMT3L or DNMT3A^R882H^–DNMT3L tetramer in a 1:1 molar ratio to allow complex formation in a buffer containing 20 mM Tris-HCl (pH 8.0), 20% glycerol and 40 mM DTT. The reaction products were sequentially purified via 100–600 mM NaCl gradient on a HiTrap Q XL column (GE Healthcare) (low salt buffer: 25 mM Tris-HCl, pH 8.0, 20 mM DTT and 100 mM NaCl; high salt buffer: 25 mM Tris-HCl, pH 8.0, 20 mM DTT and 1 M NaCl) and size-exclusion chromatography on a 16/600 Superdex 200 pg column (GE Healthcare) in buffer containing 20 mM Tris-HCl (pH 8.0), 100 mM NaCl, 0.1% β-mercaptoethanol and 5% glycerol. The final protein samples of the DNMT3A^WT^–DNMT3L–DNA and DNMT3A^R882H^–DNMT3L–DNA complexes were concentrated to 0.1 mM and used for crystallization.

### Crystallization conditions and structure determination

Crystals for DNMT3A^WT^–DNMT3L–CGA, DNMT3A^R882H^–DNMT3L–CGA and DNMT3A^R882H^–DNMT3L–CGT complexes were generated using hanging-drop vapor-diffusion method at 4 °C, from drops containing 0.2 µL of 0. 1 mM complex sample mixed with 0.8 µL of precipitant solution (0.1 M Sodium Citrate pH 4.2, 1% PEG8000). Crystals for the DNMT3A^R882H^–DNMT3L–CAG complex were generated using hanging-drop vapor-diffusion method at 4 °C, from drops containing 0.5 µL of 0.1 mM DNMT3A^R882H^–DNMT3L–CAG complex sample and 0.5 µL of precipitant solution (0.1 M Sodium Citrate pH 4.4, 0.2 % PEG8000). The crystals were soaked in cryoprotectant made of mother liquor and 35% glycerol before harvesting. X-ray diffractions datasets for the DNMT3A^R882H^–DNMT3L–CGA complex were collected on the BL12-2 beamline at the Stanford Synchrotron Radiation Light source (SSRL), SLAC National Accelerator Laboratory. X-ray diffractions datasets for the DNMT3A^WT^–DNMT3L–CGA, DNMT3A^R882H^–DNMT3L–CGT and DNMT3A^R882H^–DNMT3L–CAG complexes were collected on the beamline 5.0.1 or 5.0.2 at the Advanced Light Source (ALS), Lawrence Berkeley National Laboratory. The diffraction data were indexed, integrated, and scaled using the HKL 3000 program^43^. The structures of the complexes were solved by molecular replacement with the PHASER^44^ module in the PHNIEX software package^45^, using the structure of DNMT3A^WT^–DNMT3L–CGT DNA complex (PDB 5YX2) as search model. The structural models of the DNMT3A^WT^–DNMT3L–CGA and DNMT3A^R882H^–DNMT3L–DNA complexes were then subjected to modification using COOT^46^ and refinement using the PHENIX software package^45^ in an iterative manner. The same R-free test set was used throughout the refinement. The statistics for data collection and structural refinement of the productive covalent DNMT3A^WT^–DNMT3L–CGA and DNMT3A^R882H^–DNMT3L–DNA complexes are summarized in Supplementary Table 1.

### In vitro radioactivity-based methylation assay

In vitro methylation assay was performed in triplicates using 20-µL reactions containing 1 µM protein (either DNMT3A^WT^–DNMT3L or DNMT3A^R882H^–DNMT3L), 1 µM 36-mer DNA substrate with a single CG, CA, or CT site (5′-AATAATAATAATAATAACXATAATAATAATAATAAA-3′, X = G, A or T), 2.5 µM *S*-adenosyl-L-[methyl-^3^H]methionine with a specific activity of 18 Ci/mmol (PerkinElmer), 1.96 µM nonradioactive SAM, 50 mM Tris-HCl (pH 7.5), 0.05% β-mercaptoethanol, 5% glycerol and 200 µg/mL BSA. Reactions were left to proceed for 1 hour or indicated times at 37 °C and stopped by adding 5 µL of 10 mM nonradioactive SAM to each reaction. Samples were loaded onto DEAE membrane, washed three times with 0.2 M ammonium bicarbonate (pH 8.2), once with deionized water, and once with 95% ethanol. The membrane was left to dry for 1 hour, cut into squares to separate samples and soaked in a vial containing 4 mL of scintillation buffer. The incorporation of tritium onto DNA substrates was measured using Beckman LS6500 counter.

### Electrophoretic mobility shift assay

DNA binding analysis was conducted using 20 nM DNA duplex containing (GAC)_12_ repeats which was titrated with either DNMT3A^WT^–DNMT3L or DNMT3A^R882H^–DNMT3L, with protein concentrations ranged from 0 to 600 nM. The binding buffer contained 50 mM Tris-HCl (pH 7.5), 5% glycerol and 1 mM DTT. Samples were resolved on a 4-15% wt/v polyacrylamide gel, which was run at 4 °C using 17.8 mM Tris–borate (pH 8.3) and 0.4 mM EDTA running buffer.

### Bisulfite sequencing analysis of CpG and CpH methylation

A 626-bp long DNA substrate (denoted herein as GST3), containing 15 CpG, 38 CpA, 26 CpT, and 29 CpC sites, was amplified from one DNA fragment of the cDNA sequence encoding glutathione S-transferase gene. 0.05 µM GST3 was incubated in a methylation reaction buffer containing 400 µM SAM (S-adenosyl-L-methionine), 50 mM Tris-HCl pH 7.5, 0.05% β-mercaptoethanol and 5% glycerol in the absence or presence of either 5 µM DNMT3A^R882H^–DNMT3L for 3 h. or 1 µM WT DNMT3A^WT^–DNMT3L for 0.5 h. After incubation, the reactions were stopped by immersing samples in liquid nitrogen. The samples were subjected to bisulfite conversion using EZ DNA Methylation Gold Kit (ZYMO Research). Subsequently the upper strand of the GST3 DNA substrate was PCR amplified, cloned into pCR™4-TOPO™ TA Vector (Invitrogen), and sequenced to determine the level of CpG, CpA, CpT, and CpC methylation. The experiments were performed with two biological replicates. For replicate 1, 34 DNA clones were sequenced for methylation analysis of each protein; for replicate 2, 7 DNA clones were sequenced for methylation analysis of each protein. Comparable CpG methylation levels (20–25%) between the samples were obtained for analysis. As control, the GST3 DNA incubated in the absence of protein was subjected to bisulfite conversion, PCR amplification and cloning analysis. A bisulfite conversion rate of 98.2% was determined by the control experiment.

### ITC measurements

DNA duplex containing (GAC)_8_ repeats was used to titrate against DNMT3A^WT^-DNMT3L or DNMT3A^R882H^-DNMT3L. Both protein and DNA samples were dialyzed against the ITC buffer (20 mM Tris-HCl, pH 7.5, 100 mM NaCl, 5% glycerol) at 4 °C overnight. A MicroCal iTC200 system (GE Healthcare) was used to conduct the ITC measurements. A total of 20 injections with a spacing of 180 s and a reference power of 5 μcal/s were performed at 4 °C. The ITC curves were processed with software ORIGEN (MicroCal) using one-site fitting model.

### Reporting summary

Further information on research design is available in the Nature Research Reporting Summary linked to this article.

## Supplementary information

Supplementary Information

Reporting Summary

## Data Availability

Coordinates and structure factors for DNMT3A^WT^-DNMT3L-CGA DNA, DNMT3A^R882H^-DNMT3L-CGA DNA, DNMT3A^R882H^-DNMT3L-CGT DNA, DNMT3A^R882H^-DNMT3L-CAG DNA complexes have been deposited in the Protein Data Bank under accession codes 6W8B, 6W89, 6W8D, and 6W8J, respectively. The source data underlying Fig. 6a,c,d and Supplementary Figs 6e-h are provided as a Source Data file. Other data are available from the corresponding author upon reasonable request.

## Source data

Source Data
