# Supplementary material for: Structural basis for impairment of DNA methylation by the DNMT3A R882H mutation
Source: Nat Commun. 2020 May 8;11:2294. doi: 10.1038/s41467-020-16213-9 (PMC7210271; doi:10.1038/s41467-020-16213-9)
Supplement: Supplementary file 1 — Supplementary Information [file 41467_2020_16213_MOESM1_ESM.pdf]

Supplementary Information

Structural basis for impairment of DNA methylation by the DNMT3A R882H mutation

Hiwot Anteneh et al.

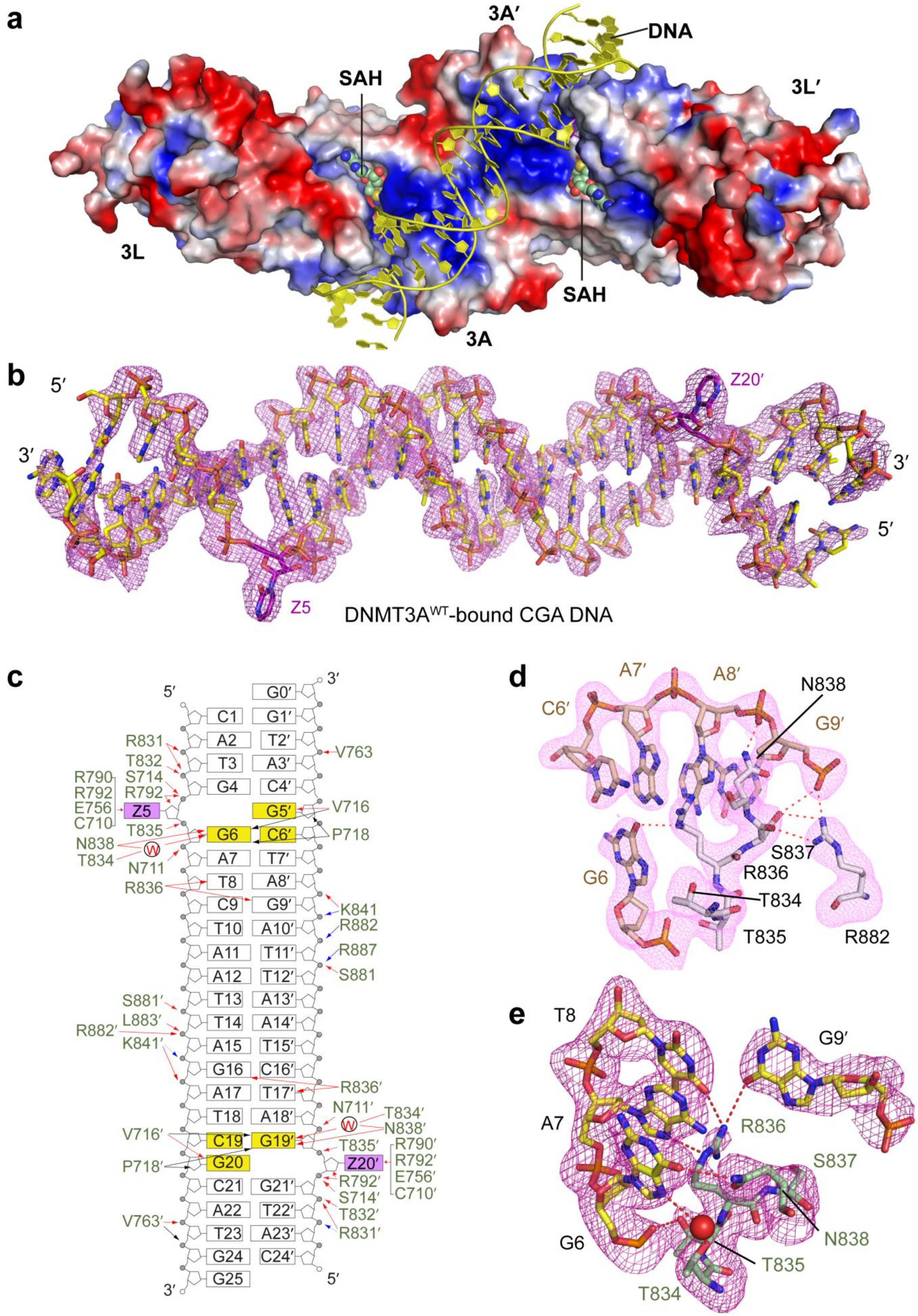
a
3A′
DNA
SAH
3L′
3L
SAH
3A
b
5′
3′
Z20′
3′
5′
Z5
DNMT3A^WT-bound CGA DNA
c
5′
3′
G0′
C1
G1′
A2
T2′
R831
V763
T3
A3′
T832
R790
S714
G4
C4′
R792
R792
E756
Z5
G5′
V716
C710
T835
G6
C6′
P718
N838
W
A7
T7′
T834
N711
T8
A8′
R836
C9
G9′
K841
T10
A10′
R882
A11
T11′
R887
A12
T12′
S881
T13
A13′
S881′
T14
A14′
L883′
R882′
A15
T15′
K841′
G16
C16′
A17
T17′
R836′
T18
A18′
N711′
T834′
W
N838′
V716′
C19
G19′
T835′
R790′
G20
Z20′
R792′
P718′
E756′
C21
G21′
R792′
C710′
S714′
A22
T22′
T832′
V763′
T23
A23′
R831′
G24
C24′
3′
G25
5′
d
A7′
A8′
N838
C6′
G9′
S837
R836
G6
T834
T835
R882
e
T8
G9′
A7
R836
S837
G6
N838
T835
T834

Supplementary Figure 1. Structural details of the DNMT3A^{WT}-CGA and DNMT3A^{WT}-CGT complexes. (a) Electrostatic surface of DNMT3A^{WT}-DNMT3L tetramer bound to CGA DNA. **(b)** Fo-Fc omit map (violet) of the CGA DNA (sticks) bound to DNMT3A^{WT}, contoured at 2.0 sigma level. **(c)** Schematic view of the DNMT3A^{WT}-DNA interactions in the DNMT3A^{WT}-CGA complex. The hydrogen-bonding, electrostatic and van der Waals interactions are indicated by red, blue and black arrows, respectively. The water-mediated hydrogen bond is indicated by letter 'W'. **(d,e)** Close-up views of the DNA interactions of the TRD loop in the DNMT3A^{WT}-CGT (d) and DNMT3A^{WT}-CGA (e) complexes. The hydrogen bonding interactions are indicated by dashed lines. The Fo-Fc omit maps (violet) for the interacting DNAs are contoured at 2.0 sigma level.

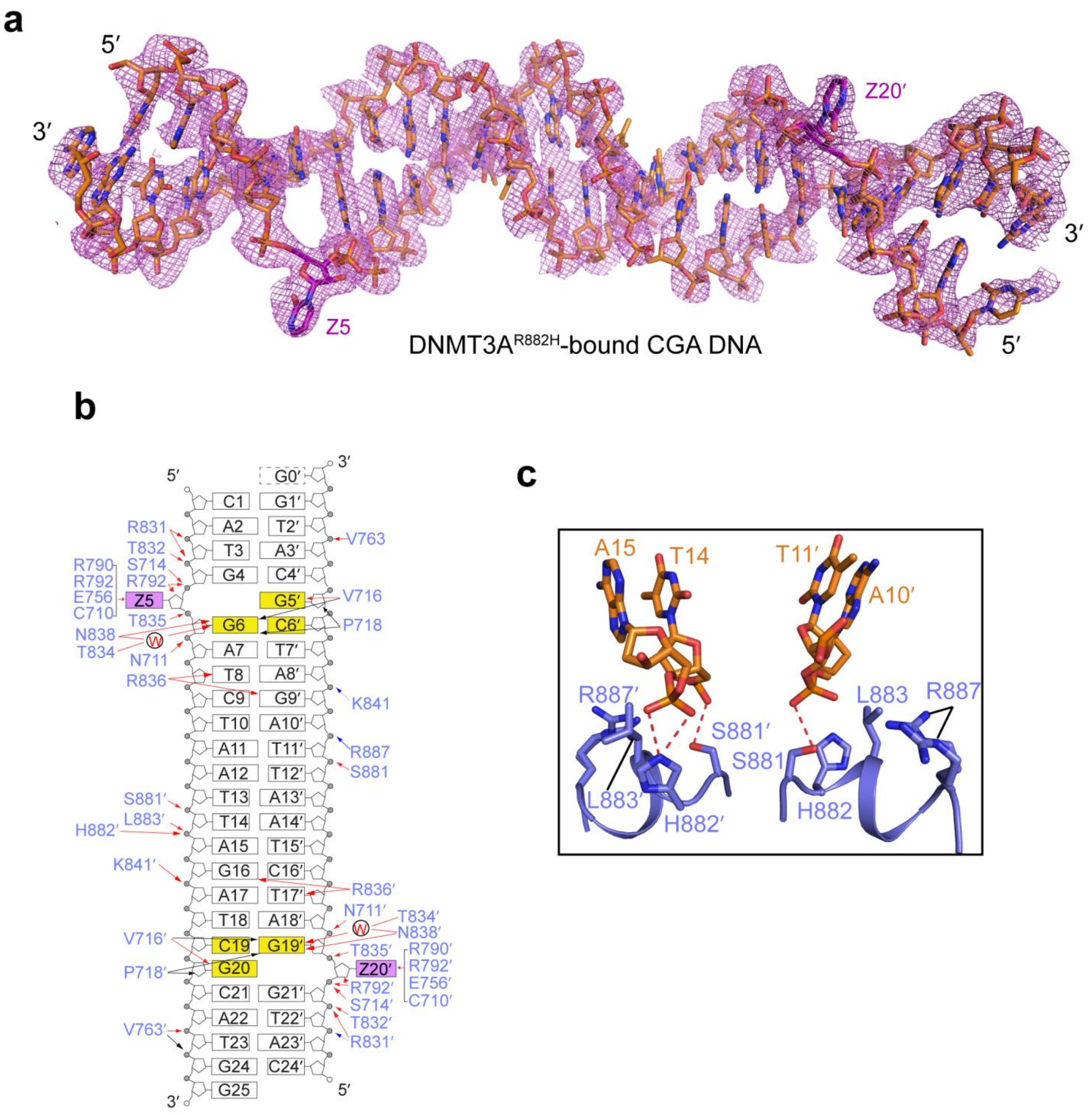

Supplementary Figure 2. Structural analysis of the DNMT3A^{R882H}-CGA complex. **(a)** Fo-Fc omit map (violet) of the CGA DNA (sticks) bound to DNMT3A^{R882H}, contoured at 2.0 sigma level. **(b)** Schematic view of the DNMT3A^{R882H}-DNA interactions in the DNMT3A^{R882H}-CGA complex. The hydrogen-bonding, electrostatic and van der Waals interactions are indicated by red, blue and black arrows, respectively. The water-mediated hydrogen bond is indicated by letter ‘W’. **(c)** Close-up view of the interactions between the RD interface and DNA in the DNMT3A^{R882H}-CGA complex. The hydrogen-bonding interactions are shown as dashed lines.

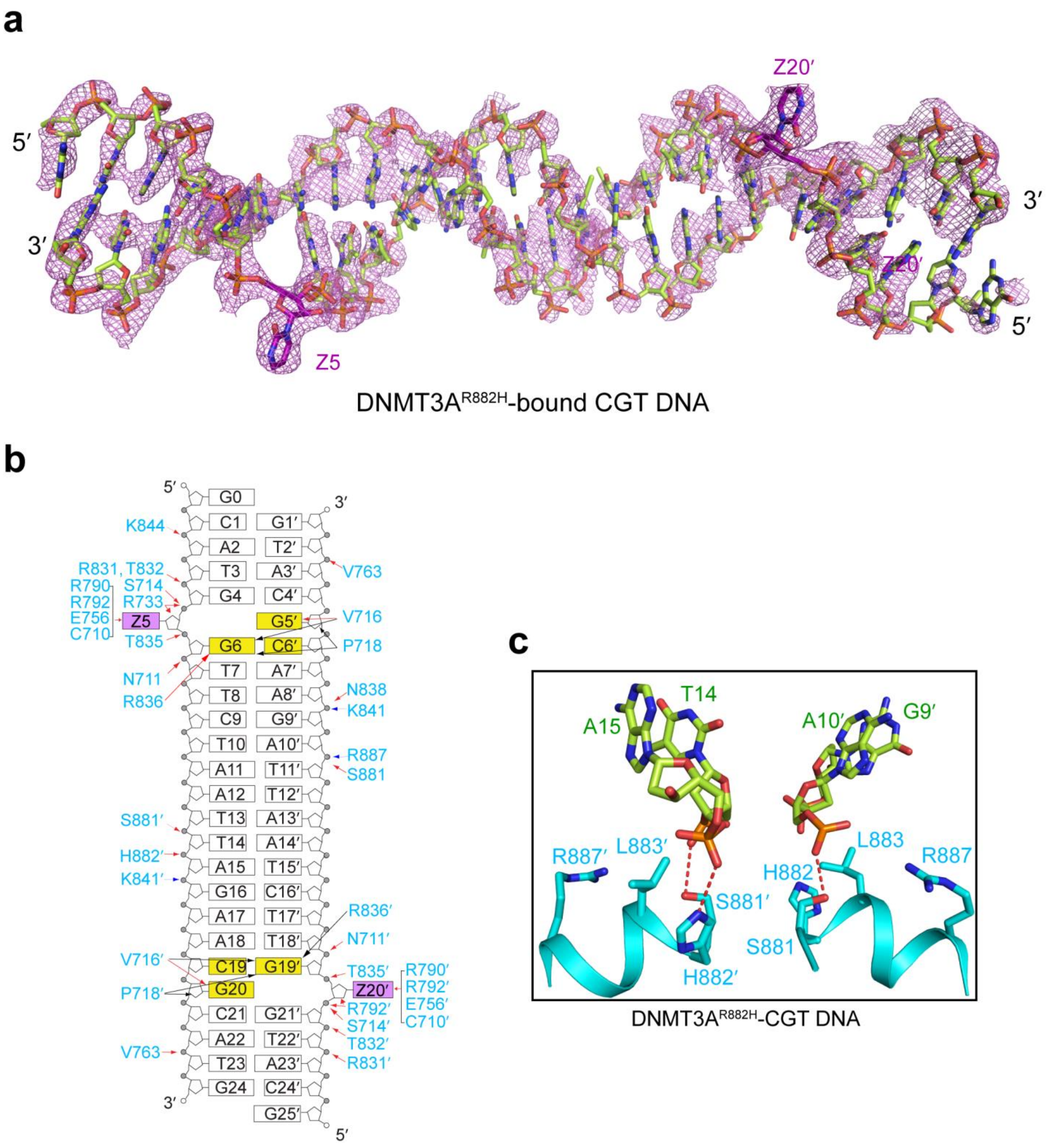

Supplementary Figure 3. Structural details of the DNMT3A^{R882H}-CGT complex. (a) Fo-Fc omit map (violet) of the CGT DNA (sticks) bound to DNMT3A^{R882H}, contoured at 2.0 sigma level. **(b)** Schematic views of the protein-DNA interactions in the DNMT3A^{R882H}-CGT complex. The hydrogen-bonding, electrostatic and van der Waals interactions are indicated by red, blue and black arrows, respectively. **(c)** Close-up view of the interaction between the RD interface and DNA in the DNMT3A^{R882H}-CGT complex. The hydrogen bonding interactions are shown as dashed lines.

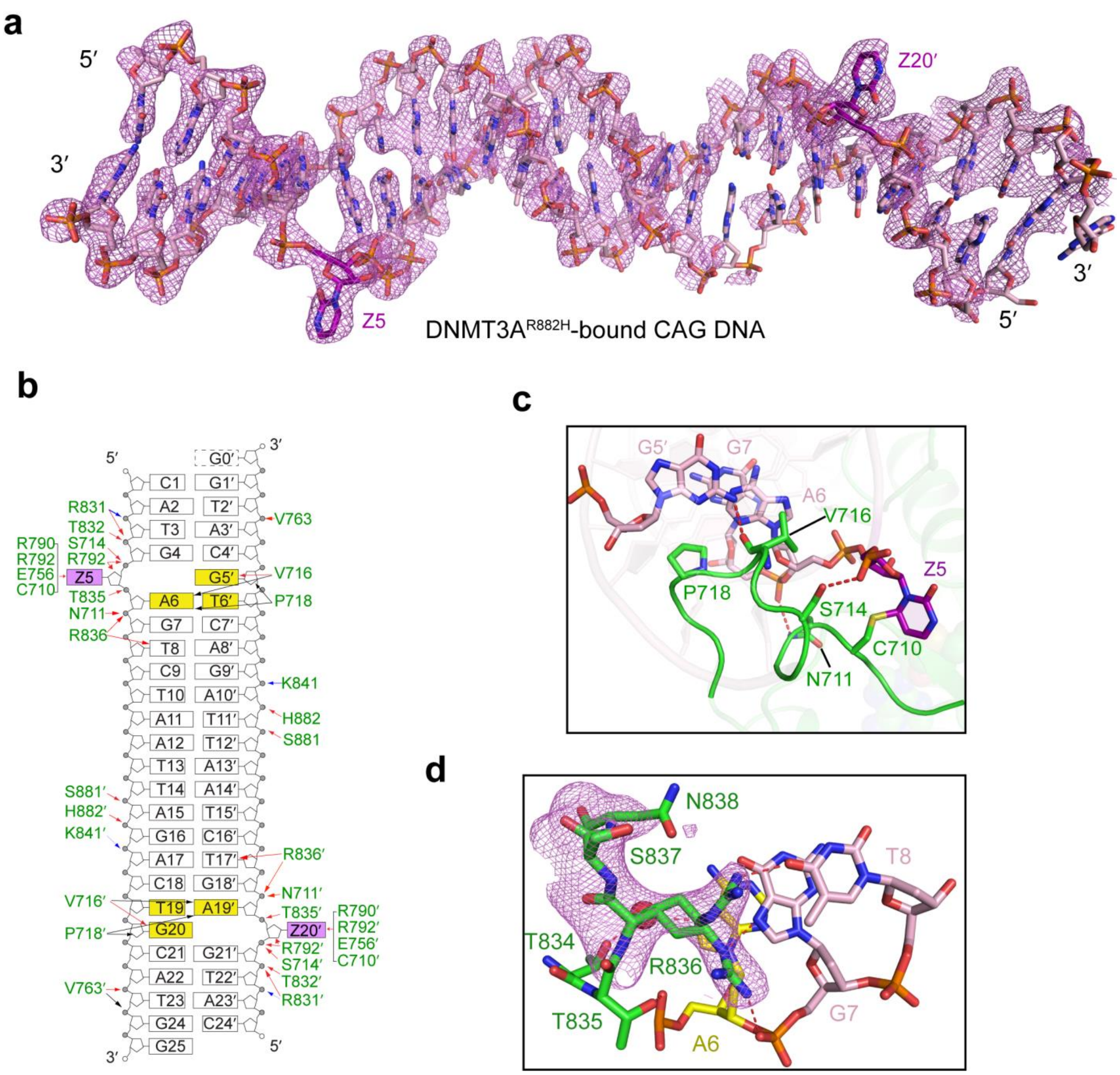

Supplementary Figure 4. Structural details of the DNMT3A^{R882H}-CAG complex. (a) Fo-Fc omit maps (violet) of the CAG DNA (sticks) bound to DNMT3A^{R882H}, contoured at 2.0 sigma level. **(b)** Schematic views of the protein-DNA interactions in the DNMT3A^{R882H}-CAG complex. The hydrogen-bonding, electrostatic and van der Waals interactions are indicated by red, blue and black arrows, respectively. **(c)** Close-up views of the interactions between the catalytic loop and DNA in the DNMT3A^{R882H}-CAG complex. **(d)** Close-up view of the interaction between the TRD loop and DNA in the DNMT3A^{R882H}-CAG complex, with the Fo-Fc omit map (violet) of residues R836-N838 contoured at 2.0 sigma level. The hydrogen bonds in (c) and (d) are shown as dashed lines. The water molecule is shown as a red sphere.

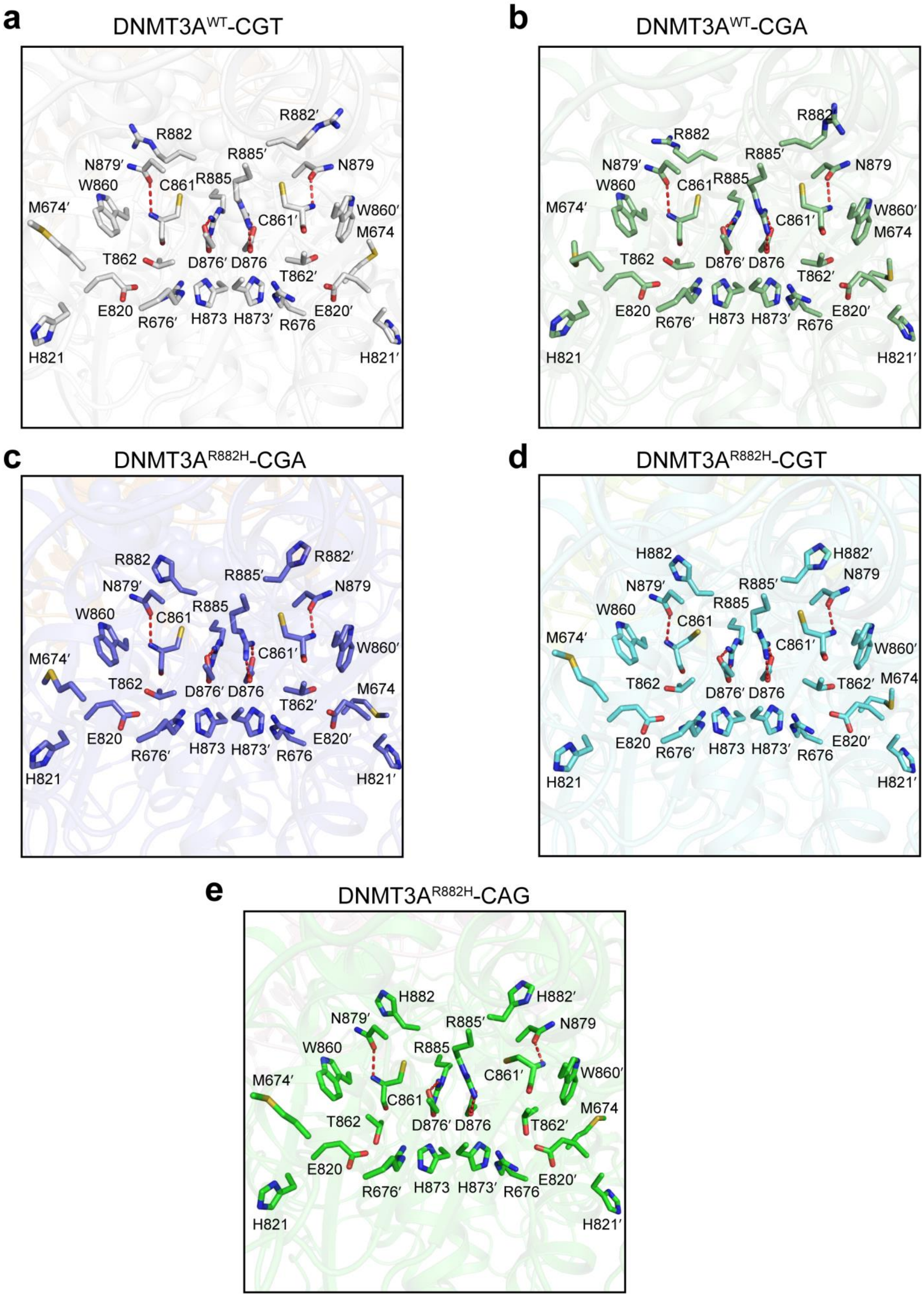
a
DNMT3A^WT-CGT
R882′
R882
R885′
N879′
N879
W860
C861
R885
C861′
W860′
M674′
M674
T862
D876′
D876
T862′
E820
R676′
H873
H873′
R676
E820′
H821
H821′
b
DNMT3A^WT-CGA
R882′
R882
R885′
N879′
N879
W860
C861
R885
C861′
W860′
M674′
M674
T862
D876′
D876
T862′
E820
R676′
H873
H873′
R676
E820′
H821
H821′
c
DNMT3A^R882H-CGA
R882
R882′
R885′
N879′
N879
R885
W860
C861
C861′
W860′
M674′
T862
D876′
D876
M674
T862′
E820
E820′
R676′
H873
H873′
R676
H821
H821′
d
DNMT3A^R882H-CGT
H882
H882′
N879
N879′
R885′
R885
W860
C861
M674′
C861′
W860′
T862
D876′
D876
T862′
M674
E820
R676′
H873
H873′
R676
E820′
H821
H821′
e
DNMT3A^R882H-CAG
H882
H882′
N879′
R885′
N879
W860
R885
C861′
W860′
M674′
C861
M674
T862
D876′
D876
T862′
E820
R676′
H873
H873′
R676
E820′
H821
H821′

Supplementary Figure 5. Intermolecular interactions at the RD interfaces of DNMT3A-DNA complexes. (a-e) Stick representations of protein residues located at the RD interfaces of the DNMT3A^{WT}-CGT (PDB 5YX2 [https://www.rcsb.org/structure/5YX2]) (a), DNMT3A^{WT}-CGA (b), DNMT3A^{R882H}-CGA (c), DNMT3A^{R882H}-CGT (d) and DNMT3A^{R882H}-CAG (e) complexes. The hydrogen bonding interactions are shown as dashed lines.

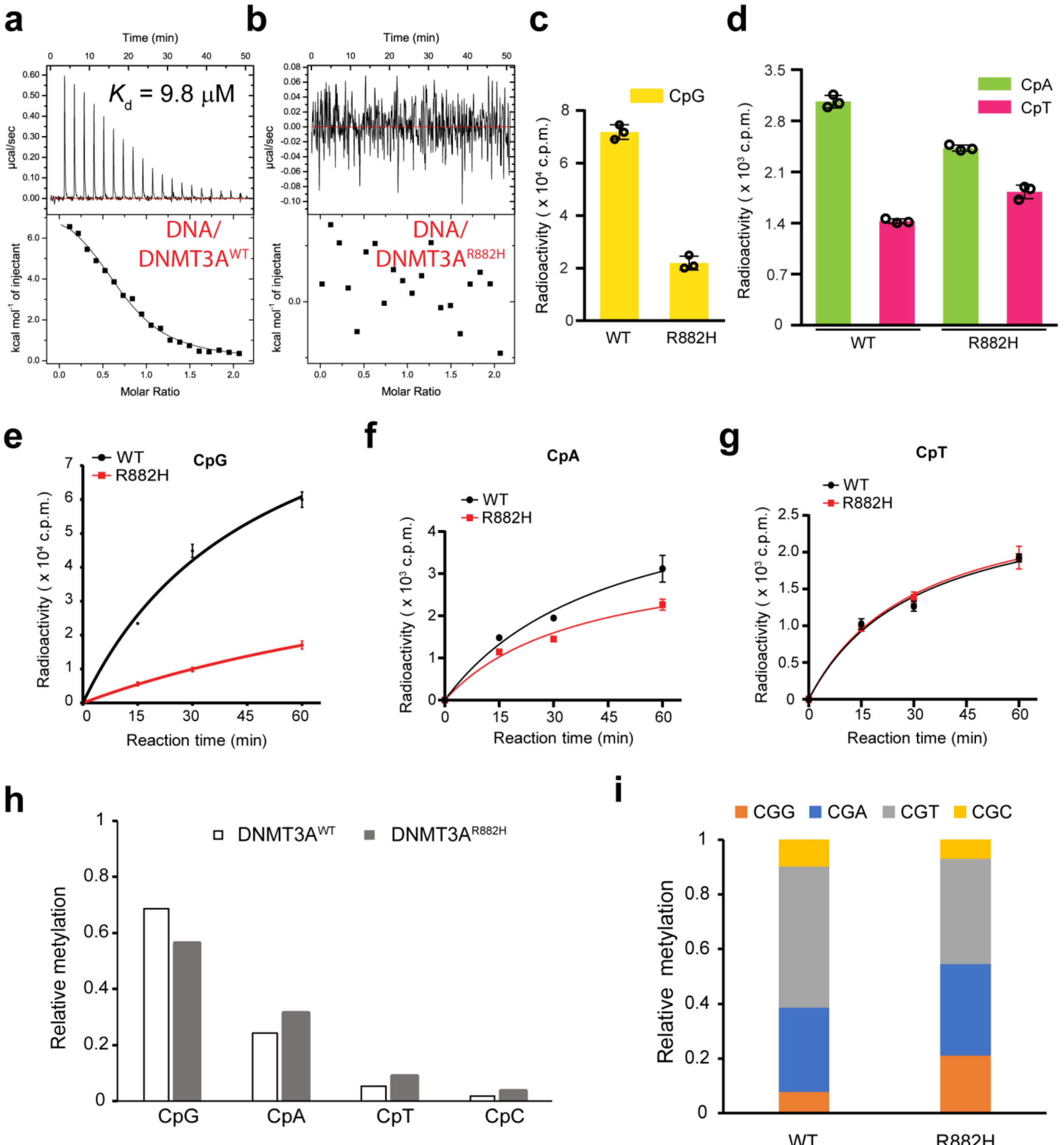

	CpG	CpA	CpT	CpC	Total
C + mC	105	266	182	203	756
mC (WT)	22	9	2	1	34
mC (R882H)	25	17	6	3	51

Supplementary Figure 6. Biochemical and enzymatic analyses of the DNMT3A^{R882H}- and DNMT3A^{WT}-mediated methylation. (a,b) Representative ITC binding curves of DNMT3A^{WT} (a) and DNMT3A^{R882H} (b) titrated against $(GAC)_8$ DNA. **(c)** *In vitro* CpG methylation of DNMT3A^{R882H}-DNMT3L and DNMT3A^{WT}-DNMT3L on a 36-

mer DNA containing single CpG site. **(d)** *In vitro* CpA and CpT methylation of DNMT3A^{R882H}-DNMT3L and DNMT3A^{WT}-DNMT3L on a 36-mer DNA containing single CpA or CpT site. **(e-g)** *In vitro* methylation kinetics of DNMT3A^{WT}-DNMT3L and DNMT3A^{R882H}-DNMT3L over (e) CpG, (f) CpA and (g) CpT DNA. The experiment in (e-g) was performed independently from (c,d). **(h)** Biological replicate of the bisulfite sequencing analysis in Figure 6d. (Top) Relative *in vitro* CpG and CpH methylation of WT and R882H-mutated DNMT3A-DNMT3L on a 626-bp DNA. The data are displayed as ratios of the methylation events of each indicated sequence context over total methylation events. (Bottom) Summary of total cytosine sites and methylation events in each indicated context, derived from bisulfite sequencing analyses of 7 clones of the 626-bp DNA incubated with WT or R882H-mutated DNMT3A-DNMT3L. Note that each DNA fragment contains 15 CpG, 38 CpA, 26 CpT and 29 CpC sites. **(i)** Relative ratio of methylation efficiency of DNMT3A^{R882H}-DNMT3L and DNMT3A^{WT}-DNMT3L for indicated sequence contexts on the 626 bp-long DNA. The experiment was repeated once with consistent results. In (c-g), n = 3 biological replicates. Data are mean ± s.d.

Supplementary Table 1. X-ray data collection and refinement statistics.

	DNMT3A^{R882H}–DNMT3L–CGT DNA (PDB: 6W8D)	DNMT3A^{R882H}–DNMT3L–CGA DNA (PDB: 6W89)	DNMT3A^{R882H}–DNMT3L–CAG DNA (PDB: 6W8J)	DNMT3A^{WT}–DNMT3L–CGA DNA (PDB: 6W8B)
**Data collection**				
Space group	H 3	P 3_2	H 3	P 3_2
Cell dimensions				
a, *b*, *c* (Å)	205.6, 205.6, 89.5	187.6, 187.6, 82.4	205.9, 205.9, 89.4	186.3, 186.3, 81.7
α, β, γ (°)	90, 90, 120	90, 90, 120	90, 90, 120	90, 90, 120
Resolution (Å)	33.16-2.60(2.69-2.60)[a]	37.72-2.50(2.59-2.50)	43.35-2.45(2.54-2.45)	46.59-2.40(2.49-2.40)
R_{merge}	0.051(1.155)	0.078(0.818)	0.112(1.496)	0.074(1.249)
$I/\sigma(I)$	13.7(0.9)	10.4(1.4)	7.8(1.9)	10.0(0.8)
$CC_{1/2}$	0.998(0.636)	0.999(0.726)	0.992(0.404)	0.999(0.644)
Completeness (%)	98.9(99.5)	98.5(93.0)	100.0(99.9)	99.9(99.9)
Redundancy	5.1(4.9)	6.7(5.9)	4.8(4.6)	5.1(4.7)
**Refinement**				
No. reflections	43,403	110,518	51,900	123,895
R_{work} / R_{free}	0.199/0.253	0.196/0.240	0.183/0.223	0.212/0.233
No. atoms				
Protein and DNA	8,238	16,730	8,330	16,745
SAH	52	104	52	104
Water	96	259	151	288
B factors (Å^2)				
DNMT3A	89.9	65.2	77.4	70.9
DNMT3L	140.2	124.0	125.9	130.8
DNA	158.4	120.0	129.8	116.8
SAH	89.1	60.1	80.6	66.6
Water	88.5	68.8	83.2	68.1
r.m.s deviations				
Bond lengths (Å)	0.004	0.006	0.004	0.004
Bond angles (°)	0.703	0.968	0.708	0.675

[a]Values in parentheses are for highest-resolution shell. Each structure was determined using the dataset collected from a single crystal.

Supplementary Table 2. Summary of primer sequences.

Name	Sequence	Notes
DNMT3A(R882H)_F	CAATATGAGCCATCTGGCACGTCAGCGT	DNMT3A R882H mutation
DNMT3A(R882H)_R	TGCCAGATGGCTCATATTGCTAACATCGG	DNMT3A R882H mutation
DNMT3A_MTase_F	CGCGGCGGTCTCGGATCCGCAGAAAAACGTAAACCGATTCGTG	Cloning of DNMT3A MTase domain into pRSFDuet-1
DNMT3A_MTase_R	GGCGGCGCGGCCGCTTAAACGCAGGCAAAATATTC	Cloning of DNMT3A MTase domain into pRSFDuet-1
DNMT3L_F	CGCGGCCATATGATGTTCGAAACCGTGCCTGTG TGG	Cloning of DNMT3L C-terminal domain into pRSFDuet-1
DNMT3L_R	CGCGGCGGTCTCCTCGAGTTATAAAGAGGAAGTGAGTTCTG	Cloning of DNMT3L C-terminal domain into pRSFDuet-1
GST3-F	TTGAAGAAAAATATGAAGAGGATTTGTATGAG	Bisulfite sequencing analysis
GST3-R	CCCCTCCAACACAACTTCC	Bisulfite sequencing analysis
