# Supplementary material for: Structural basis for impairment of DNA methylation by the DNMT3A R882H mutation
Source: Nat Commun. 2020 May 8;11:2294. doi: 10.1038/s41467-020-16213-9 (PMC7210271; doi:10.1038/s41467-020-16213-9)
Supplement: Supplementary file 2 — Reporting Summary [file 41467_2020_16213_MOESM2_ESM.pdf]

nature research

Corresponding author(s): Jikui Song

Last updated by author(s): Apr 6, 2020

Reporting Summary

Nature Research wishes to improve the reproducibility of the work that we publish. This form provides structure for consistency and transparency in reporting. For further information on Nature Research policies, see Authors & Referees and the Editorial Policy Checklist .

Statistics

For all statistical analyses, confirm that the following items are present in the figure legend, table legend, main text, or Methods section.

n/a	Confirmed	
☐	☒	The exact sample size (*n*) for each experimental group/condition, given as a discrete number and unit of measurement
☐	☒	A statement on whether measurements were taken from distinct samples or whether the same sample was measured repeatedly
☒	☐	The statistical test(s) used AND whether they are one- or two-sided<br>*Only common tests should be described solely by name; describe more complex techniques in the Methods section.*
☒	☐	A description of all covariates tested
☒	☐	A description of any assumptions or corrections, such as tests of normality and adjustment for multiple comparisons
☐	☒	A full description of the statistical parameters including central tendency (e.g. means) or other basic estimates (e.g. regression coefficient) AND variation (e.g. standard deviation) or associated estimates of uncertainty (e.g. confidence intervals)
☒	☐	For null hypothesis testing, the test statistic (e.g. *F*, *t*, *r*) with confidence intervals, effect sizes, degrees of freedom and *P* value noted<br>*Give P values as exact values whenever suitable.*
☒	☐	For Bayesian analysis, information on the choice of priors and Markov chain Monte Carlo settings
☒	☐	For hierarchical and complex designs, identification of the appropriate level for tests and full reporting of outcomes
☒	☐	Estimates of effect sizes (e.g. Cohen's *d*, Pearson's *r*), indicating how they were calculated

Our web collection on statistics for biologists contains articles on many of the points above.

Software and code

Policy information about availability of computer code

Data collection	X-ray diffraction data were collected on synchrotron beamline 5.01 and 5.0.2, at the Advanced Light Source (ALS), Lawrence Berkeley National Laboratory, and beamline 12-2 at the Stanford Synchrotron Radiation Light source (SSRL), SLAC National Accelerator Laboratory.
Data analysis	HKL3000, Phenix.refine and Phaser-MR modules in PHENIX 1.15.2-3472-000, WinCoot 0.8.9 and Pymol v0.99 softwares were used for X-ray diffraction data processing and analysis. Origin 7.0 was used for ITC data fitting analysis.

For manuscripts utilizing custom algorithms or software that are central to the research but not yet described in published literature, software must be made available to editors/reviewers. We strongly encourage code deposition in a community repository (e.g. GitHub). See the Nature Research guidelines for submitting code & software for further information.

Data

Policy information about availability of data

All manuscripts must include a data availability statement. This statement should provide the following information, where applicable:

- Accession codes, unique identifiers, or web links for publicly available datasets
- A list of figures that have associated raw data
- A description of any restrictions on data availability

The X-ray structures (coordinates and structure factor files) for DNMT3A(WT)-DNMT3L-CGA DNA, DNMT3A(R882H)-DNMT3L-CGA DNA, DNMT3A(R882H)-DNMT3L-CGT DNA, DNMT3A(R882H)-DNMT3L-CAG DNA complexes have been deposited in the Protein Data Bank under accession codes 6W8B, 6W89, 6W8D and 6W8J, respectively. The source data underlying Figs 6a,c,d and Supplementary Figs 6e-h are provided as a Source Data file.

Field-specific reporting

Please select the one below that is the best fit for your research. If you are not sure, read the appropriate sections before making your selection.

☒ Life sciences ☐ Behavioural & social sciences ☐ Ecological, evolutionary & environmental sciences

For a reference copy of the document with all sections, see nature.com/documents/nr-reporting-summary-flat.pdf

Life sciences study design

All studies must disclose on these points even when the disclosure is negative.

Sample size	Biochemical and enzymatic assays were completed using wild-type DNMT3A-DNMT3L and R882H-mutated DNMT3A-DNMT3L
Data exclusions	None.
Replication	Bisulfite sequencing and ITC analyses were repeated once with consistent results. The enzymatic assays were derived from three independent measurements, which gave consistent results.
Randomization	The biochemical and enzymatic assays require a rational approach for activity comparison. Therefore, randomization is not applicable to our experimental set up.
Blinding	Blinding is not applicable to any biochemical assay performed in this study.

Reporting for specific materials, systems and methods

We require information from authors about some types of materials, experimental systems and methods used in many studies. Here, indicate whether each material, system or method listed is relevant to your study. If you are not sure if a list item applies to your research, read the appropriate section before selecting a response.

Materials & experimental systems

n/a	Involved in the study	
☒	☐	Antibodies
☒	☐	Eukaryotic cell lines
☒	☐	Palaeontology
☒	☐	Animals and other organisms
☒	☐	Human research participants
☒	☐	Clinical data

Methods

n/a	Involved in the study	
☒	☐	ChIP-seq
☒	☐	Flow cytometry
☒	☐	MRI-based neuroimaging
